# Natural Fibrous Materials Based on Fungal Mycelium Hyphae as Porous Supports for Shape-Stable Phase-Change Composites

**DOI:** 10.3390/polym15234504

**Published:** 2023-11-23

**Authors:** Adeliya R. Sayfutdinova, Kirill A. Cherednichenko, Maria A. Rakitina, Valeria N. Dubinich, Kristina A. Bardina, Maria I. Rubtsova, Daria A. Petrova, Vladimir A. Vinokurov, Denis V. Voronin

**Affiliations:** Department of Physical and Colloid Chemistry, National University of Oil and Gas “Gubkin University”, Moscow 119991, Russia

**Keywords:** porous support, biopolymers, mycelium, *Trametes hirsuta*, microfibrillar cellulose, composites, phase-change materials, stearic acid, eicosane

## Abstract

Adsorption of organic phase-change materials (PCMs) by the porous matrix of microfibrillar cellulose (MFC) is a simple and versatile way to prepare shape-stable phase-change composites, which are promising as sustainable thermoregulating additives to construction materials. However, due to MFC inherent morphology, the resulting composites have relatively low poured density that complicates their introduction in sufficient amounts, for instance, into mortar mixes. Unlike MFC, fungal mycelium has, by an order, less fibrils thickness and, thus, possesses significantly higher poured density. Herein, we studied the feasibility of fungal mycelium-based matrices as alternative biopolymeric porous supports for preparation of sustainable and shape-stable phase-change composites. Two methods were employed to prepare the porous mycelium-based supports. The first one was the solid-state fermentation, which resulted in partial biotransformation of MFCs to mycelium hyphae, while the second one was the liquid-state surface fermentation, used to cultivate the reference matrix of *Trametes hirsuta* hyphae. The phase-change composites were prepared by adsorption of model organic PCMs on porous biopolymer matrices. The mass ratio of support/PCM was 40/60 wt%. The composites were studied with respect to their structure, composition, poured density, latent heat storage properties, and thermal and shape stability. The employment of the partially transformed to mycelium-hyphae MFC fibers was found to be a suitable way to prepare phase-change composites with improved poured density while preserving a reasonable latent heat capacity and shape stability as compared to the MFC/PCM composites.

## 1. Introduction

Adsorption of organic phase-change materials (PCMs) onto porous support prepared with modified and unmodified microfibrillar cellulose (MFC) was shown to be an effective way to produce shape-stable phase-change composites [1]. This technique has a number of benefits like ease of preparation, no need for harsh synthesis conditions, and tunable latent heat storage properties of the resulting composites. In addition, cellulose is the most abundant biopolymer on Earth, with a vast base of renewable sources, including agricultural residues and household waste, that can be processed into MFC through a simple mechanical treatment [2,3]. At the same time, organic PCMs like fatty acids can be acquired from the renewable sources as well [4]. It makes MFC-based phase-change composites an attractive sustainable additive to construction materials to enhance their thermoregulating performance. However, due to extensive MFC structure, the MFC-based composites have low poured density, which means a relatively low mass fraction of the composites that can be added, e.g., to dry mortar mixes. For instance, the preliminary studies demonstrated that only 10 wt% of the composite containing 40 wt% of MFC can be embedded into the mortar mix [5]. The mass fraction of the composite could be increased to 15 wt% by reducing the MFC content in the composition to 30 wt%; however, this was accompanied by less shape stability and PCM leakage. Thus, it is of interest to find novel biopolymer substrates to obtain sustainable phase-change composites with good shape stability, high latent heat capacity, and reasonable poured density that will be suitable for enhancing the thermoregulating properties of the conventional construction materials.

A way to improve the poured density of the composites is to employ the supporting material with a morphology different to MFC. A possible option is further treatment of MFC to nanofibrillar cellulose (NFC). Ordinary, NFC has a width of 5–60 nm and a length of several µm. However, the significant drawback of this approach is that the further disintegration of MFC requires sufficient energy supply (over 25,000 kWh per ton of the final NFC) and additional pretreatment of MFC in harsh conditions, including acid hydrolysis, enzymatic degradation, or TEMPO-oxidation [6]. Alternatively, the self-growing biopolymeric supports can be employed. From this point, a porous matrix of fungal mycelium appears to be a promising biopolymeric support in certain respects. Mycelium is a vegetative part of fungi. This is a self-growing material consisting of thin fibers called hyphae. Hyphae entangle together into a fibrous network to assemble a flexible supporting biopolymeric matrix [7]. Due to the large number of functional groups on the cell wall of hyphae, such as hydroxyl and amine, mycelial fibers are promising for the development of new functional biobased materials [8]. Nowadays, mycelium is a highly tunable, biodegradable, low-cost material with a broad spectrum of exploitation [9], including packaging [10,11,12], construction [13,14,15,16,17], and biomedical applications [18,19]. Considering the support for PCM composites, the mycelial hyphae have an order less fiber thickness compared to MFCs that should result in higher bulk density. Additionally, unlike cellulose fibrils, mycelial hyphae are self-growing fibers with low energy demand for cultivation, they do not form byproducts, and may use organic waste for their nutrition. Last, but not least, the resulting fibers do not require a mechanical treatment to be isolated.

In our previous study, we demonstrated the approach for cultivation of fungi strains employing cellulose microfibrils simultaneously as a feeding substrate and as a template for mycelium growth [20]. This resulted in direct biotransformation of MFCs to mycelium hyphae. With this approach, we evaluated the growth rate of various fungi strains and found out that *Ganoderma lucidum* and *Trametes hirsuta* revealed the most prominent rate of MFC biotransformation along with the optimal fibrous structure of the mycelium. Thus, the aim of this study was to evaluate the feasibility of mycelium as a porous biopolymeric support to prepare shape-stable phase-change composites by the adsorption of different types of organic PCMs (paraffin and fatty acid). To achieve this, three types of biopolymer supports were employed: initial MFCs, partly transformed support consisting of MFCs and mycelium (MFC–MYC), and mycelium hyphae (MYC), combined with PCMs at the same mass ratio. The evaluation was carried out with respect to the structure, poured density, shape and thermal stability, thermal reliability, and latent heat storage properties of the resulting phase-change composites. To our best knowledge, the phase-change composites with mycelium-based supporting matrices have not been studied before.

## 2. Materials and Methods

### 2.1. Materials

Raw cellulose pulp was supplied by Arkhangelsk Pulp and Paper Mill (Novodvinsk, Russia). Stearic acid (SA), KH_2_PO_4_, K_2_HPO_4_, and MgSO_4_ were purchased from Sigma-Aldrich (St. Louis, MO, USA). Eicosane was purchased from Acros Organics (Gell, Belgium). The commercially available unhopped malt extract was purchased from a local supplier in Russia. Enzymatic peptone and agar were purchased from Becton Dickinson and Company (Franklin Lakes, NJ, USA). Glucose was purchased from PanReac AppliChem (Barcelona, Spain) and yeast extract was purchased from Bio Springer (Maisons-Alfort, France).

### 2.2. Methods

#### 2.2.1. Preparation of Microfibrillar cellulose

Microfibrillar cellulose (MFC) was prepared through deep mechanical treatment of cellulose pulp according to the procedures described previously [21,22]. In particular, 5 wt% aqueous suspension of cellulose pulp was prepared and sequentially treated for 5 times with a Masuko Supermasscolloider grinder (Masuko Sangyo, Kawaguchi, Japan) at 6000 rpm. The resulting MFC slurry was dried in a drying chamber (Binder, Tuttlingen, Germany) at 60 °C until the mass leveled off. Prior to use, the dry MFC was homogenized with Anton Paar BM500 ball mill (Anton Paar, Ostfildern, Germany) at 30 Hz for 3 min.

#### 2.2.2. Strain and Culture Conditions

Basidiomycete *Trametes hirsuta* MT-17.24 was obtained from the collection of basidiomycete fungi strains of the Mycotechnology Laboratory of the Department of Physical and Colloidal Chemistry (Gubkin University). It was maintained in a 100 mm Petri dish with malt agar (30 g/L of unhopped malt extract, 3 g/L of enzymatic peptone, and 15 g/L of agar) as a growth medium, transferring the culture to fresh medium every 30 days.

##### Solid-State Fermentation of Composite Mycelium/Cellulose Fibers

The method was proposed in [23]. MFC was used as a carbon source, whereas peptone was employed as a nitrogen source. First, homogenized MFC was distributed in a thin layer over the surface of the Petri dishes. After this, peptone mixed with distilled water was added to fulfill MFC/peptone and MFC/water ratios of 10 to 1 and 1 to 5, respectively. Then, the sterilization was performed for 45 min at 1.5 atm and 121 °C. After the substrate was cooled to room temperature, 3 × 3 mm agar fragments with *T. hirsuta* mycelium were placed in the center of the Petri dishes. The Petri dishes were incubated in a thermostat at 28 °C for two weeks. The produced composite mycelium/cellulose fibers (MFC–MYC) were separated and dried at 60 °C overnight. Prior to use, the dried MFC–MYC mass was homogenized with the ball mill as well.

##### Liquid-State Surface Fermentation of Pure Mycelium Fibers

The matrix of mycelium hyphae (MYC) was grown at the liquid–air interface in static conditions. The liquid culture medium used for cultivation consisted of glucose (15 g/L), peptone (2.5 g/L), yeast extract (3 g/L), and KH_2_PO_4_ (1 g/L), K_2_HPO_4_ (0.2 g/L), and MgSO_4_ (0.5 g/L) salts, as was reported in [24]. The medium was sterilized for 45 min at 1.5 atm and 121 °C and poured into Petri dishes. After the medium was cooled to room temperature, 3 × 3 mm agar fragments with *T. hirsuta* mycelium were placed in the center of the dishes. The samples were incubated in a thermostat at 28 °C for three weeks. Upon cultivation, the mycelium was separated and dried at 60 °C overnight. Prior to use, the dried MYC mass was homogenized with the ball mill, as described previously.

#### 2.2.3. Preparation of Phase-Change Composites

The phase-change composites were prepared by addition of the supporting fibers to melted PCMs with a support/PCM mass ratio of 40/60 wt%. Stearic acid (SA) and eicosane (E) were chosen as representative fatty acid and paraffin PCMs. The required mass of PCMs was melted in a glass beaker. After this, a suitable amount of the supporting fibers was added and thoroughly stirred for 5 min. The resulting mixtures were left at 3 °C for 30 min to let the deposited PCM crystalize and form bulk composites. After that, the formed bulk materials were mechanically treated to restore the fibrous structure of the chosen supports. Table 1 describes the composition of all obtained samples.

#### 2.2.4. Characterization

The surface morphology of the bare fibers and phase-change composites was studied with a JIB-4501 (JEOL, (Akishima, Tokyo, Japan) scanning electron microscope (SEM) at an accelerating voltage of 10 kV. The fibers and composites were placed on a conductive carbon tape and sputtered with a 10 nm Au layer for effective charge sink.

Chemical composition of the bare fibers and phase-change composites were studied with Fourier transformed infrared (FTIR) spectroscopy. The spectra were acquired in absorbance mode with a Nicolet iS 10 FTIR Spectrometer equipped with germanium ATR crystal (Thermo Scientific, Waltham, MA, USA). The wavenumber range was 4000–600 cm^−1^. The final spectra were a result of averaging of 16 iterative scans.

The poured density of the bare fibers and the phase-change composites was evaluated with respect to the mass of the predefined volume of the samples. The bare fibers or the composites were placed into graduated glass tubes, avoiding excessive compacting. After this, the different volumes of the samples were weighed, and the poured density of the different volumes was calculated. The measurements were carried out three times. The resulting poured density was defined as an averaged density calculated for different volumes of the samples.

Differential scanning calorimetry (DSC) was used to measure the latent heat storage properties and thermal reliability of the phase-change composites. The measurements were performed with a DSC214 Polyma calorimeter (Netzsch, Selb, Germany) in the range of 20 to 80 °C for the composites prepared with SA and in the range of 10 to 60 °C for the composites prepared with eicosane. The heating/cooling rate was 10 °C per min. Upon measurements, the acquired heating and cooling curves were time-integrated to figure out the melting and crystallization enthalpies of PCMs in the composites structure as an area of endothermic and exothermic peaks.

The thermal decomposition patterns of bare fibers, PCMs, and phase-change composites were studied with thermogravimetric analysis (TGA). The decomposition patterns were recorded with an STA 449 F5 Jupiter (Netzsch, Selb, Germany) device in the range of 30 to 800 °C with a ramp of 10 °C per min. The samples mass varied from 5 to 35 mg, depending on the substrate type. The measurements were performed under a nitrogen atmosphere.

The shape stability of the phase-change composites was evaluated with respect to the leakage rate of PCMs from the composite structure [1]. The samples were placed on filter paper and heated in a drying box at the temperature of 5 °C above the end of melting of PCMs according to DSC: 60 °C and 75 °C for the composites prepared with eicosane and SA, respectively. The composites were heated for 10 h, whereas the mass measurements were performed every hour. The PCM leakage was evaluated as the relative mass loss by the composites.

## 3. Results and Discussions

### 3.1. Preparation and Characterization of the Mycelium-Based Fibers

**Figure 1:**
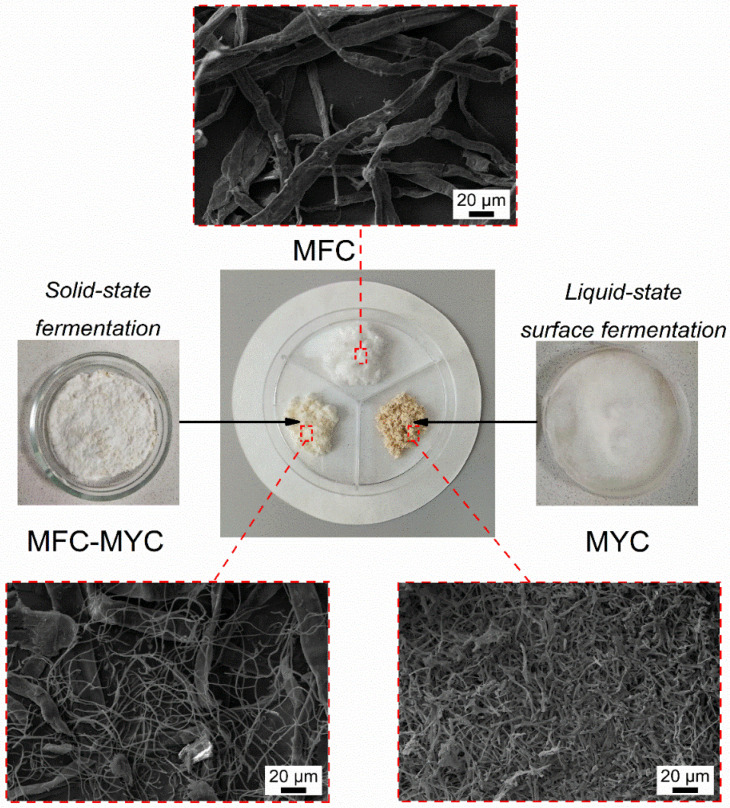
The schematic representation of the preparation methods of mycelium-based fibrous supports and their structure at the macroscopic and microscopic scale.

Two types of fungal mycelium-based porous supports were employed for PCM deposition, with MFC as a reference support. Figure 1 shows an overview of the preparation methods along with the macro- and microstructure of the resulting supporting fibers. The reference MFC was ribbon-like fibers with an average longitudinal size of 10–20 µm.

In the preparation of the MFC–MYC sample, the aim was to obtain individual mycelial hyphae (along with cellulose fibers) with a narrow distribution of longitudinal size, avoiding the formation of a fungal skin. Ordinarily, in the solid-state fermentation, the hyphae at the air/solid interface (aerial hyphae) tend to grow faster than those in the substrate volume (substrate hyphae) [25]. This is related to the formation of the mycelium shell on the substrate surface, limiting the oxygen access to the substrate hyphae. As a result, the growth rate of mycelium in the substrate volume is slowed down, which reduces the overall rate of biotransformation eventually. It was demonstrated previously that the mycelium growth over the whole substrate can be facilitated by increasing the incubation time, controlling the sufficient oxygen content in solid medium, and by supplying growth promoters [26,27]. Thus, in cultivation of MFC–MYC fibers, the thin layers of MFC substrates were prepared and placed at the bottom of the Petri dishes to reach the homogeneous mycelium growth. As the growth of hyphae occurred at the air/solid interface due to direct oxygen access, the increase in surface-to-volume ratio of the substrates resulted in a uniform biotransformation of MFC with the low degree of mycelium maturation. Furthermore, by this approach, the growth of mycelium can be easily controlled and interrupted at the desired stage. Additionally, it was reported that the homogenization of the substrate during the incubation facilitates the uniform hyphae growth and distribution over the substrate [28,29]. For this reason, the substrates were additionally gently homogenized after a week of incubation and set for further mycelium growth. The SEM images of MFC–MYC fibers (Figure 1) revealed both cellulose microfibrils and fungi hyphae with the longitudinal size of 1–1.5 µm, which confirmed the successful partial transformation of MFC to mycelium during the solid-state fermentation.

Another type of mycelium-based substrate studied in this work was the mycelium hyphae, which were self-grown at the liquid/air interface under static conditions, employing liquid-state surface fermentation [18,30,31]. The resulting substrate was a mycelial mat with a high density of branched fungal hyphae. The part of the mycelial mat that was in contact with the liquid can be recognized as yellow-colored compact hyphae, while the upper part that was in contact with the air was represented as aerial white hyphae [32]. Since the aerial hyphae at the air interface were still growing, the bottom layer of the mycelial film was developed under almost anaerobic conditions [33].

The macrostructure of the prepared substrates was also different in appearance and morphology (Figure 1). MFC and MFC–MYC were reminiscent of cotton-like fibers, yet the MFC–MYC fibers had a slight mushroom odor and a light brown color. In contrast, the MYC fibers were more like a powder of brown color with a strong mushroom odor. It should be mentioned that the initial mycelium-based fibers produced by both solid-state cultivation and liquid-state surface cultivation possess white color; however, they tend to become brown at the drying stage. The color change in mycelium-based fibers is associated with a nonenzymic reaction that is known as a Maillard or carbonyl-amine reaction [34,35]. The color change during drying is typical for all fungal-based materials [26].

### 3.2. Preparation and Study of the Phase-Change Composites

The feasibility of mycelium-based substrates for preparation of shape-stable phase-change composites was studied, employing two types of organic PCMs, which are stearic acid and eicosane. The composites were prepared at 40/60 wt% of substrate to PCM ratio, as was previously demonstrated as an optimal composition for preparation of stable phase-change composites using MFC as a supporting material [1].

**Figure 2:**
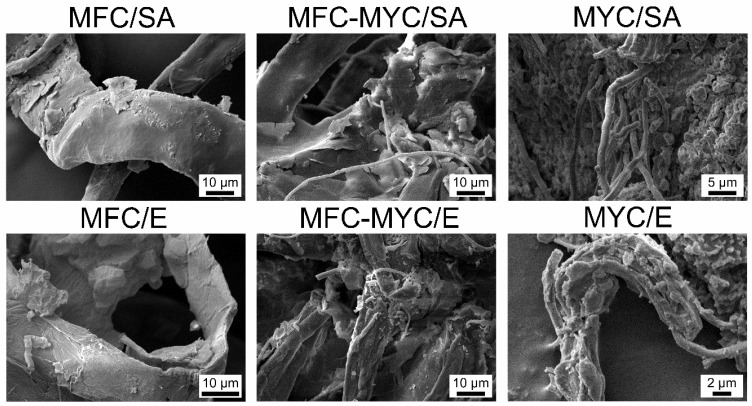
SEM micrographs of the phase-change composites prepared by deposition of stearic acid (SA) and eicosane (E) on the MFC, MFC–MYC, and MYC fibers.

Figure 2 shows the SEM images of the resulting composites prepared with different types of PCMs and supporting fibers. The successful deposition of PCMs can be recognized as a change of fiber morphology due to appearance of the lamellar surface layer. In MFC-based composites, the PCMs appeared as a more or less uniform layer covering the fiber surface. In composites prepared with MFC–MYC fibers, the PCMs appeared as a surface layer on the MFC as well. However, one may recognize that the bundles of hyphae not only adsorbed PCMs as a surface layer but also captured bulk pieces of PCMs within their volume. The same can be seen in the composites prepared with MYC fibers, where the hyphae were mostly embedded in bulk PCM pieces.

**Figure 3:**
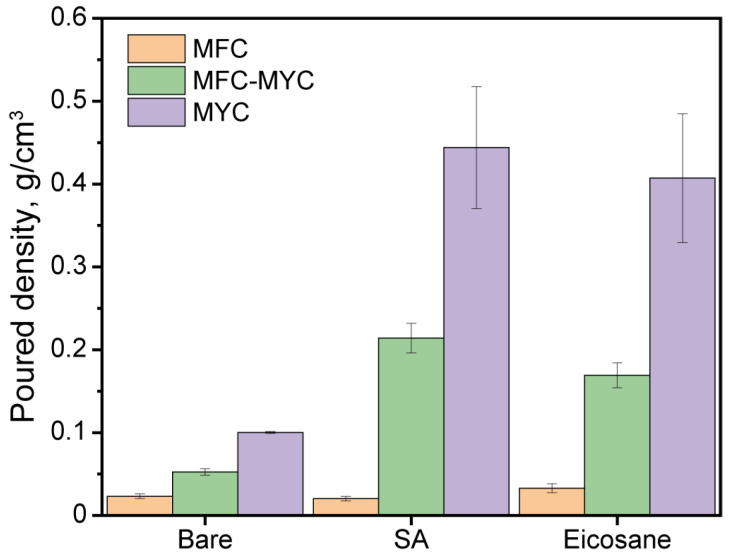
The poured density of the bare fibrous substrates and the phase-change composites prepared by the deposition of SA and eicosane on the MFC, MFC–MYC, and MYC fibers.

Figure 3 shows the results of the evaluation of the poured density of the bare fibers and phase-change composites. Considering the bare fibers, the poured density gradually increased with the content of mycelium hyphae in the fibers’ structure. This suggests a denser packing of the fibers with the decrease in the longitudinal size. The poured density of MFC, MFC–MYC, and MYC was found to be 0.023 ± 0.003 g/cm^3^, 0.053 ± 0.003 g/cm^3^, and 0.100 ± 0.001 g/cm^3^, respectively. Noticeably, the poured density of MFC–MYC fiber was 2.3 times higher compared to initial MFC. This implies that the newly developed mycelium hyphae effectively filled the gaps in the MFC structure. The obtained density values are in agreement with the SEM images, demonstrating the different morphology of the bare fibers. Further, the phase-change composites prepared by the deposition of PCMs onto MFC fibers demonstrated almost the same poured density as bare MFC (0.020 ± 0.003 g/cm^3^ for MFC/SA and 0.033 ± 0.005 g/cm^3^ for MFC/E). According to SEM images, the PCMs were deposited mostly over the MFC surface and almost no bulk pieces of PCM remained in the composite structure. In turn, the poured density of the composites prepared by deposition of PCMs onto MFC–MYC fibers was three to four times higher than that of the bare MFC–MYC fibers (0.214 ± 0.017 g/cm^3^ for MFC–MYC/SA and 0.169 ± 0.015 g/cm^3^ for MFC–MYC/E). Such an increase can be explained by the additional adsorption of PCMs within the volume of hyphae bundles. Analogously, the composites prepared by deposition of PCMs onto MYC fibers had a poured density four times higher than the bare MYC fibers (0.444 ± 0.074 g/cm^3^ for MYC/SA and 0.407 ± 0.078 g/cm^3^ for MYC/E).

**Figure 4:**
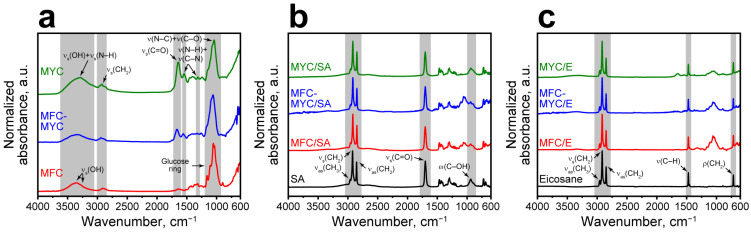
(**a**) FTIR spectra of the MFC, MFC–MYC, and MYC fibers; (**b**) FTIR spectra of MFC/SA, MFC–MYC/SA, and MYC/SA composites; (**c**) FTIR spectra of MFC/E, MFC–MYC/E, and MYC/E composites.

The chemical structure of the bare fibers and phase-change composites was studied with FTIR spectroscopy. Originally, cellulose and fungal mycelium have a different chemical nature. Cellulose is a polysaccharide composed of D-glucose units linked with ß-glycosidic bonds. Mycelium has a more complicated structure. In addition to α- and ß-glucans, fungal cell walls also contain lipids, proteins, and chitin/chitin–glucan complex. Therefore, the bands corresponding to hydroxyl, carbonyl, and amide groups appeared in the spectra of mycelium-based fibers (Figure 4a). The following absorption bands were inherent to all fiber types: the band between 3000 and 3600 cm^−1^ corresponds to stretching vibrations of OH groups; the band around 2855 cm^−1^ refers to symmetric stretching of CH_2_ group. The low intensity band at 1645 cm^−1^ in FTIR spectrum of MFC relates to the OH vibrations, whereas the band at 1650 cm^−1^ in mycelium-based fibers refers to the stretching vibrations of the C=O bonds of the carbonyl group. The intense band in the range of 1150–920 cm^−1^ observed in all spectra is derived from the pyranose ring oscillations. Additionally, the FTIR spectra of mycelium-based fibers demonstrated maximums of absorption that are not presented in the MFC spectrum. The most prominent ones are the bands at 1543, 1313, and 1245 cm^−1^ due to vibration of N–H and C–N bonds in chitin [18,30,36]. Since cellulose is not fully biotransformed, the intensity of these bands in the spectrum of MFC–MYC fibers is lower than those in the spectrum of the pure mycelium fibers.

SA can be recognized by absorption maximums due to antisymmetric stretching of the CH_2_ group at 2915 cm^−1^ and 2849 cm^−1^, antisymmetric stretching of the CH_3_ group at 2954 cm^−1^, and stretching of the C=O bond at 1700 cm^−1^ (Figure 4b). Analogously to SA, eicosane has adsorption maximums related to the stretching of CH_2_ and CH_3_ groups in the 2954–2849 cm^−1^ range, peaks due to stretching vibration of the CH group at 1470 cm^−1^, and rocking vibration of the CH_2_ group at 717 cm^−1^ of the paraffin chain (Figure 4c). The FTIR spectra of the obtained phase-change composites revealed adsorption maximums of the corresponding PCM and support without any shift and addition of new bands. It confirms the hypothesis of PCM physical adsorption without any covalent bonding.

**Figure 5:**
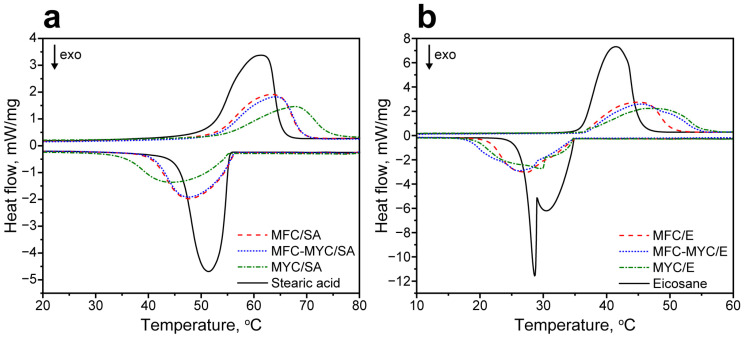
(**a**) Melting and crystallization curves of the initial SA and MFC/SA, MFC–MYC/SA, and MYC/SA composites; (**b**) melting and crystallization curves of the initial eicosane and MFC/E, MFC–MYC/E, and MYC/E composites.

Latent heat storage properties of the phase-change composites were evaluated with DSC. Figure 5a shows the comparison of melting and crystallization curves of the pristine SA and the composites prepared by its adsorption on the supporting porous matrix. SA demonstrated a single endothermic peak with a peak melting temperature T_M_ = 61 °C and melting enthalpy ΔH_M_ = 178 J/g [37]. In MFC/SA and MFC–MYC/SA composites, the melting point slightly shifted to 63–64 °C, while the ΔH_M_ reduced to 105–106 J/g, proportional to the mass content of the adsorbed PCM. In contrast, the MYC/SA composite demonstrated a more pronounced shift in melting point to 68 °C. Apparently, this can be related to a different conformation of SA in the composite structure compared to MFC/SA and MFC–MYC/SA composites. The ΔH_M_ of MYC/SA composite also reduced with respect to initial SA to 95 J/g. The crystallization curve of SA had a single exothermic peak with a peak crystallization temperature T_C_ = 51 °C and crystallization enthalpy ΔH_C_ = 182 J/g [37]. Analogously to melting, MFC/SA and MFC–MYC/SA composites demonstrated slightly shifted crystallization points, T_C_ = 48 °C, and ΔH_C_ reduced to 106–109 J/g. The crystallization point of SA in MYC/SA composite was shifted even further toward T_C_ = 44 °C, while ΔH_C_ was 97 J/g. Considering the melting and crystallization enthalpy values in SA-containing composites, one can calculate the actual SA content as follows:

(1) $$E_{a}=\frac{∆H_{M,comp}+∆H_{C,comp}}{{∆H}_{M,PCM}+{∆H}_{C,PCM}}\times 100\%$$

where Δ*H_M_*_,*comp*_ and Δ*H_C_*_,*comp*_ are the melting and crystallization enthalpies of SA in the composite, while Δ*H_M_*_,*PCM*_ and Δ*H_C_*_,*PCM*_ are the melting and crystallization enthalpies of the pure SA [38]. According to Equation (1), *E_a_* was calculated to be 59%, 59%, and 55% for MFC/SA, MFC–MYC/SA, and MYC/SA composites, respectively. In turn, the theoretical SA content can be calculated as follows:

(2) $$E_{t}=\frac{m_{PCM}}{m_{PCM}+m_{M}}\times 100\%$$

where *m_PCM_* is the mass of the initially added SA, and *m_M_* is the mass of the employed supporting material for SA adsorption [39]. Thus, the loading efficiency of SA in the composites can be figured out as follows [40]:

(3) $$LE=\frac{E_{a}}{E_{t}}\times 100\%$$

Considering this, the loading efficiency of SA in MFC/SA and MFC–MYC/SA composites was 98%, and 92% in MYC/SA composite.

Figure 5b shows the comparison of melting and crystallization curves of the pristine eicosane and the composites prepared by its adsorption. The melting curve of eicosane had a single endothermic peak with T_M_ = 41 °C and ΔH_M_ = 236 J/g [41]. The melting curves of the composites also demonstrated a single melting peak. Similar to the SA-containing composites, the melting points were slightly shifted in MFC/E and MFC–MYC/E composites to 45 °C, whereas the MYC/E composite demonstrated a further shift of T_M_ to 48 °C. The ΔH_M_ of the composites was also reduced to 137–139 J/g, proportional to eicosane content. The crystallization curve of eicosane had two exothermic peaks with peak crystallization points at 30 °C and 28 °C, which is typical for even long-chained n-alkanes [42]. These two peaks correspond to formation of the metastable rotator R_I_ phase and its further transition to the stable triclinic phase in alkanes during crystallization [43,44]. The overall ΔH_C_ of eicosane crystallization was 236 J/g. The MFC/E and MFC–MYC/E composites demonstrated a single crystallization peak with T_C_ = 26–27 °C. This can be attributed to the formation of a thin eicosane layer over the fiber surface. Compared to the bulk eicosane, the thin layer has a much higher surface-to-volume ratio. This led to a more significant contribution of surface crystallization phenomena to the overall crystallization process. As a result, this led to primary formation of the crystalline phase, and only minor shoulders appeared in the crystallization curve due to the formation of the rotator phase as compared to bulk eicosane [42]. The overall enthalpy of eicosane crystallization in MFC/E and MFC–MYC/E composites was 136 J/g and 143 J/g, respectively. In contrast, the crystallization curve of the MYC/E composite demonstrated a more pronounced shoulder due to the formation of the rotator phase and a more evident peak due to the formation of the crystalline phase. This can be attributed to the presence of the bulk eicosane in the composite structure along with the one adsorbed on the MYC fibers. The ΔH_C_ of eicosane crystallization in the MYC/E composite was 140 J/g. According to Equation (1), the actual eicosane content was 57% in the MFC/E composite, 59% in the MFC–MYC composite, and 58% in the MYC/E composite. This gives a loading efficiency of eicosane in the composites of 97%, 98%, and 98% respectively.

**Figure 6:**
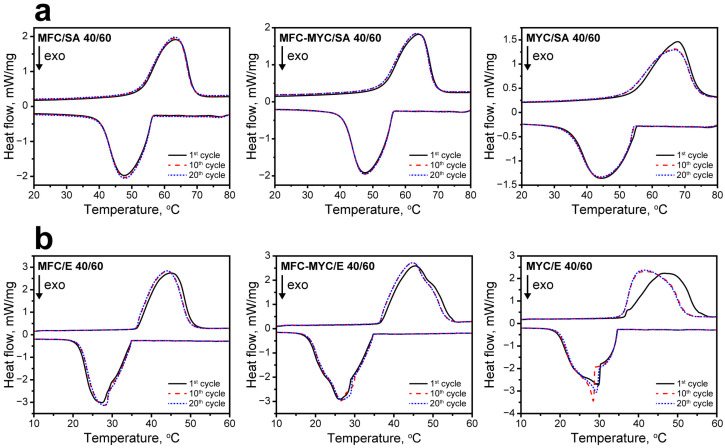
(**a**) The DSC curves of MFC/SA, MFC–MYC/SA, and MYC/SA composites acquired after the 1st, 10th, and 20th heating/cooling cycles; (**b**) the DSC curves of MFC/E, MFC–MYC/E, and MYC/E composites acquired after the 1st, 10th, and 20th heating/cooling cycles.

The reliability of the latent heat storage and release properties of the composites was studied with cyclic DSC measurements. Figure 6 shows the melting and crystallization curves acquired after the 1st, 10th, and 20th heating/cooling cycles. Overall, the most evident changes in the shape of the endothermic and exothermic peaks occurred after the first heating/cooling cycle in all compositions. This can be explained by the change in the conformation of PCMs in the composite structure after the first heating cycle [1,45]. Thereafter, the composites demonstrated negligible changes in latent heat storage and release behavior, as can be seen from a comparison of initial melting and crystallization curves and the curves acquired after the 10th and 20th heating/cooling cycles. The most obvious change in the shape of endothermic and exothermic peaks was demonstrated by MYC/SA and MYC/E composites. Apparently, this can be related to the presence of the sufficient fraction of bulk PCMs in their structure. After the first melting, the PCMs were rearranged in the composite structure, and after this, they melted and crystallized in the same way in every further heating/cooling cycle. The comparison of phase transition temperatures and enthalpies of PCMs in the composites after the 1st and 20th heating/cooling cycles is given in Table 2.

**Figure 7:**
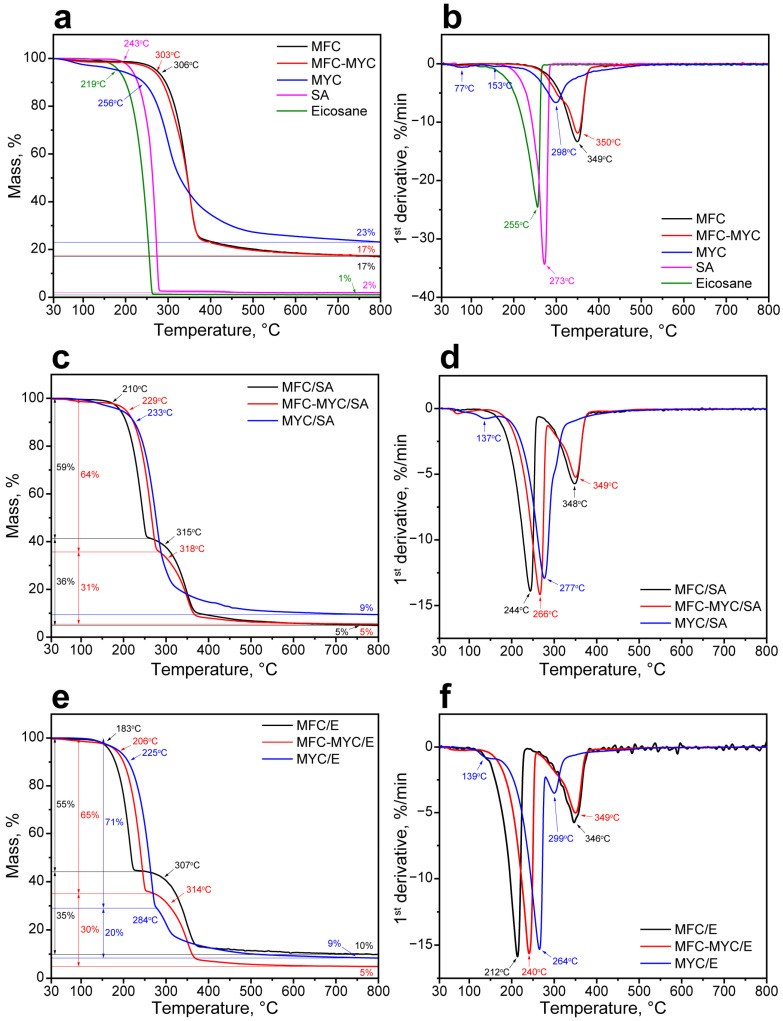
(**a**) TGA curves of MFC, MFC–MYC, and MYC fibers along with TGA curves of SA and eicosane; (**b**) DTGA curves of MFC, MFC–MYC, and MYC fibers along with DTGA curves of SA and eicosane; (**c**) TGA curves of MFC/SA, MFC–MYC/SA, and MYC/SA composites; (**d**) DTGA curves of MFC/SA, MFC–MYC/SA, and MYC/SA composites; (**e**) TGA curves of MFC/E, MFC–MYC/E, and MYC/E composites; (**f**) DTGA curves of MFC/E, MFC–MYC/E, and MYC/E composites.

The thermal stability of the composites was studied with TGA. At first, thermal decomposition patterns of the supporting fibers and PCMs were acquired (Figure 7). The decomposition of MFC, MFC–MYC, and MYC fibers occurred in one stage. Furthermore, MFC and MFC–MYC demonstrated almost similar decomposition behavior with only slight differences in onset decomposition temperatures, which were 306 °C and 303 °C respectively (Figure 7a). The main decomposition of these fibers occurred between 310 °C and 375 °C and can be attributed to the random rupture of glycosidic linkages in cellulose macromolecules [46]. The DTGA curves revealed the same maximum rate of decomposition temperatures (MRDTs) of 350 °C, yet the curve of MFC–MYC fibers had a minor shoulder at around 300 °C that can be attributed to decomposition of β-glucans in mycelium components [31] (Figure 7b). This suggests that the decomposition of MFC–MYC fibers was mainly defined by the decomposition of MFC in its structure. Unlike cellulose, fungal mycelium has a more complex chemical structure, in accordance with the FTIR data. Thus, the decomposition of MYC fibers involved several sequential studies, visualized as a gradual mass loss over the measured temperature range. In the beginning, the mass loss was due to evaporation of the absorbed water at 30–110 °C [47]. This stage can be also recognized by the peak at 77 °C in the DTGA curve. The next stage at 110–256 °C can be attributed to decomposition of α-glucans with an MRDT of 153 °C [48]. The major mass loss that occurred in the 256–350 °C range corresponded to decomposition of β-glucans with an MRDT of 298 °C [49]. The final stage was due to the decomposition of residual chitin–glucans and chitin in the 350–500 °C range that appeared as a peak extending towards higher temperatures in the DTGA curve [46]. In turn, SA and eicosane decomposed in one stage (Figure 7a,b). SA started to decompose at 243 °C with an MRDT at 273 °C and completely lost its weight at 285 °C. In turn, eicosane started to decompose at 219 °C with an MRDT at 255 °C and the end of decomposition at 264 °C.

The phase-change composites had more complex thermal degradation patterns owing to sequential decomposition of PCMs and porous supports in their structure. Figure 7c,d show TGA and DTGA curves of MFC/SA, MFC–MYC/SA, and MYC/SA composites. The decomposition of the MFC/SA composite took two prominent stages, related to decomposition of SA followed by decomposition of MFC. SA started to decompose at 210 °C and reached the maximum rate of decomposition at 244 °C. Compared to bulk SA, the SA adsorbed on the MFC surface demonstrated reduced thermal stability that is explained by an increase in its specific surface area [1]. It is known that the thermal degradation rate constant tends to increase along with a specific surface area of the material; thus, the transition from a bulk to a thin-layered structure results in a decreased onset temperature and reduced thermal stability [50]. The weight loss at this stage was 59%, which is in good agreement with the theoretical and actual SA content in the composite calculated from DSC data. The second stage in MFC/SA decomposition corresponded to the decomposition of MFC. This was confirmed by the same MRDT of 348 °C as for initial MFC. The MFC–MYC/SA composite demonstrated a similar decomposition pattern but with less prominent stages. SA adsorbed on MFC–MYC fibers had better thermal stability, with increased onset decomposition temperature of 229 °C and MRDT of 266 °C, as compared to SA/MFC composite. This can be related to the presence of bulk SA fraction captured by MYC fibers. The mass loss after the first decomposition step was 64%, which is a bit higher than theoretical and calculated actual SA content. This can be explained by the simultaneous decomposition of SA and mycelium components. The second stage of MFC–MYC/SA decomposition was due to decomposition of MFC, as evidenced by an MRDT of 349 °C. In contrast, the MYC/SA composite demonstrated one main decomposition stage that started at 233 °C, although a minor peak can be seen in the DTGA curve at 137 °C. This suggests that the decomposition of the composite was initiated by decomposition of α-glucans in mycelium followed by simultaneous decomposition of SA (as can be seen by MRDT of 277 °C) and β-glucans (as evidenced by a minor extension in the 300–330 °C range) and completed by decomposition of chitin–glucan and chitin components that appeared as peak tailing beyond 330 °C on the DTGA curve. In this composite, it was hard to distinguish a particular mass due to SA decomposition because of the similar temperature range to β-glucans degradation. Noticeably, the onset and maximum rate of decomposition temperatures of SA were shifted towards the higher temperature range, which can be explained by the sufficient presence of the bulk SA in the composite.

The same was true for the phase-change composites prepared with eicosane, except the decomposition stages were more apparent owing to lower onset temperature and MRDT of eicosane compared to SA (Figure 7e,f). The decomposition of the MFC/E composite started at 183 °C with an MRDT of 212 °C. At this stage, the mass loss was 55%, which is in agreement with an actual eicosane content figured out from DSC. The second stage of decomposition started at 307 °C with an MRDT of 346 °C, which corresponds to the MFC decomposition pattern. Analogously to the SA/MFC composite, eicosane adsorbed onto the MFC surface demonstrated a reduced onset and maximum rate of decomposition temperatures. The MFC–MYC/E composite started to decompose at 206 °C with an MRDT of 240 °C. The mass loss at this stage was 65% due to additional decomposition of mycelium components. The second stage started at 314 °C with an MRDT of 349 °C and was also related to MFC degradation. In the MYC/E composite, four stages can be defined in the decomposition pattern. The first one was due to the initiation of α-glucans degradation, which can be recognized by the minor peak at 139 °C on the DTGA curve. The most prominent impact was due to the decomposition of eicosane, which started at 225 °C with an MRDT of 264 °C. Similar to the MYC/SA composite, eicosane adsorbed on the matrix of mycelium hyphae demonstrated better thermal stability compared to eicosane adsorbed on MFC and MFC–MYC fibers. The third stage was due to the decomposition of β-glucans, which can be recognized by the peak at 299 °C on the DTGA curve. The final stage was the decomposition of chitin–glucan and chitin components, as evidenced by extending the peak beyond 330 °C. It should be noted that despite the composites demonstrating reduced thermal stability compared to pristine PCMs, they remained fairly stable in the temperature range of their potential application, which is defined by their melting and crystallization points.

**Figure 8:**
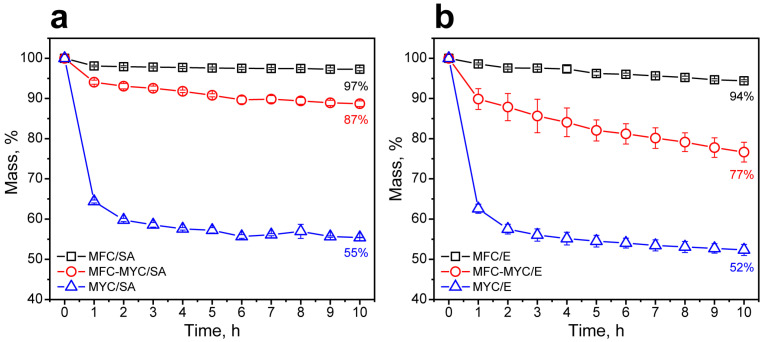
(**a**) The mass loss by MFC/SA, MFC–MYC/SA, and MYC/SA composites in 10 h of heating at 75 °C; (**b**) the mass loss by MFC/E, MFC–MYC/E, and MYC/E composites in 10 h of heating at 60 °C.

The shape stability of the composites was evaluated by measuring the composite mass during continuous heating on the absorbing material (cellulose paper filter) at a temperature above the melting point derived from the melting curves. Figure 8a shows the mass loss by the composites containing SA. The MFC/SA composite demonstrated high shape stability with a mass loss of only 3% in 10 h of heating at 75 °C. The most apparent mass loss was in 1 h of heating, which can be related to the leakage of free SA pieces from the composite. This is in good agreement with the results on the study of the shape stability of the MFC/SA 40/60 composite that we obtained in our previous work under similar conditions [1]. On one hand, the adsorption of SA on the MFC partially transformed to mycelium hyphae led to a more prominent mass loss of 13% at the same time. Analogously to the MFC/SA composite, the most apparent mass loss was observed in 1 h of heating. However, the mass of the MFC–MYC/SA composite kept gradually reducing further. This can be related to the leakage of the bulk SA captured by the bundles of mycelium hyphae. Finally, the MYC/SA composite demonstrated the most significant mass loss. This could be due to the massive leakage of the bulk SA entrapped by the hypha bundles. The mass loss in 1 h of heating was 37%, whereas the overall mass loss in 10 h was 45%. This confirms the suggestion that at an MYC/SA ratio of 40/60 wt%, most of the SA was absorbed in the volume of mycelium hypha bundles and only a minor fraction was spread over the hypha surface. The SA that was not interacting with the fiber surface was free to leak in the liquid state, while the SA adsorbed on the hypha surface remained stable. The same tendency was found for the composites prepared with eicosane in 10 h of heating at 60 °C (Figure 8b). The only difference was that the eicosane was even further prone to leakage due to the absence of the functional groups in its structure that may facilitate its interaction with the supporting fibers.

**Figure 9:**
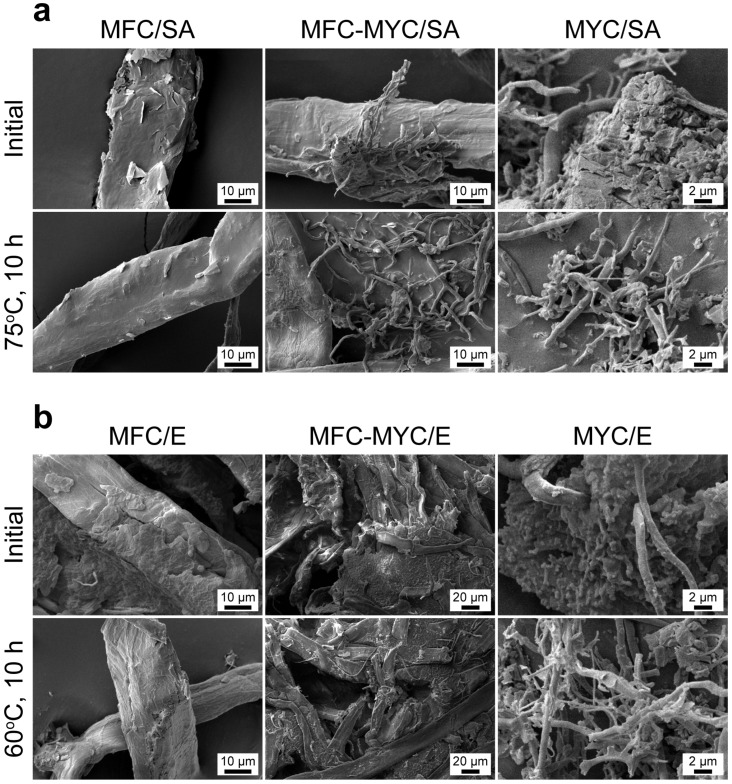
(**a**) SEM images of MFC/SA, MFC–MYC/SA, and MYC/SA composites before and after 10 h of heating at 75 °C; (**b**) SEM images of MFC/E, MFC–MYC/E, and MYC/E composites before and after 10 h of heating at 60 °C.

After the leakage test, the structure of the phase-change composites was studied with SEM. Figure 9a shows the SEM images of MFC/SA, MFC–MYC/SA, and MYC/SA composites before and after 10 h of heating at 75 °C. In the MFC/SA composite, the multiple melting and crystallization of SA on the MFC surface resulted in the homogenization of the SA layer and more uniform covering of the fibers. The change in conformation of the SA layer explains the minor shift of the endothermic peak on the melting curve after the first heating cycle through the cyclic DSC measurements. In the MFC–MYC/SA composite, SA remained on the MFC surface while the MYC fibers were set free, as SA had leaked from the MYC bundles, although it can be seen that MYC fibers captured some of the SA droplets and prevented their leakage as well. The massive leakage of SA from the MYC/SA composite also resulted in the appearance of the separate MYC/SA fibers. The same behavior was demonstrated by MFC/E, MFC–MYC/E, and MYC/E composites (Figure 9b). In the MFC/E composite, the initial eicosane layer appeared nonuniform, which can be related to the less-firm coupling of eicosane with the MFC surface, resulting in its more pronounced disturbance due to mechanical treatment compared to SA. However, after heating at 60 °C, the covering of MFC with eicosane appeared more uniform owing to the redistribution of the melted eicosane over the MFC surface. In the MFC–MYC/E composite, the heating was accompanied by the leakage of eicosane absorbed in the composite volume. Finally, the massive leakage of eicosane from the MYC/E composite resulted in the formation of separate composite fibers.

Considering the SEM images and results of the leakage test, it can be concluded that some residual mass of PCMs remained on the MYC surface (about 15% in MYC/SA composite and about 12% in MYC/E composite). Taking into account the TGA data that demonstrated that the main decomposition of MYC fibers started at a higher temperature, the mass loss in these composites could be related only to the leakage of PCMs. Thus, with complete PCM leakage, the mass of the composites should reduce to 40%, corresponding to the bare MYC fibers. With respect to the remaining mass of SA and eicosane, this suggests that the optimal proportion to prepare the shape-stable fibers should be 73/27 wt% for the MYC/SA composite and 77/23 wt% for the MYC/E composite. Under the same considerations, the optimal mass ratio for the composites prepared with MFC–MYC fibers should be 46/54 wt% for the MFC–MYC/SA composite and 52/48 wt% for the MFC–MYC/E composite.

Furthermore, summarizing the data on the structural and thermal properties of the prepared composites, it appears that the bigger fibers were capable of retaining the thicker PCM layer. That is why, under the same substrate/PCM ratio of 40/60 wt%, the PCMs were mostly adsorbed on the MFC surface and absorbed into the volume of MYC bundles. This explains the increase in the thermal stability of the composites along with the growth of MYC fraction in the supporting fibers and, therefore, with the growth in the bulk PCM content. Additionally, the change in the conformation of the PCMs that did not interact with the fiber surface may explain the shift in the shape and position of the endothermic peaks in the DSC curves.

Overall, the introduction of MYC fibers seems a reasonable improvement in preparation of phase-change fibers, although the adjustment of the substrate/PCM ratio has to be performed to obtain the shape-stable structures. Even considering that the mass fraction of PCMs need to be reduced by 2–3 times if employing the MYC fibers, the poured density of these composites was more than 10 times higher than that of MFC-based composites. This should give an overall improvement in latent heat capacity if the MYC-composites will be added to the building mixes. On the other hand, the composites prepared by the adsorption of PCMs onto MFC–MYC porous support demonstrated relatively high latent heat capacity and reasonable reduction in shape stability. Their composition needs to be only slightly adjusted. Furthermore, these composites demonstrated a more than five times higher poured density compared to MFC-based composites. This makes MFC–MYC/PCM composites a promising additive to construction materials. Thus, further studies will be devoted to the preparation of the shape-stable phase-change composites by adsorption of PCMs on MFC–MYC and MYC fibers and studying their feasibility as thermoregulating additives to dry building mixes.

## 4. Conclusions

In this work, microfibrillar cellulose (MFC), mycelium hyphae (MYC), and microfibrillar cellulose partly transformed to hyphae (MFC–MYC) were studied as porous supporting substrates for preparation of shape-stable phase-change composites by the adsorption of stearic acid and eicosane with a support/PCM ratio of 40/60 wt%. Previously, this mass ratio was found to be optimal for the preparation of the shape-stable composite fibers by the deposition of stearic acid onto MFC fibers with the longitudinal size of 10–15 µm. The MFC–MYC fibers were prepared through the solid-state fermentation technique, which involved the partial transformation of cellulose microfibrils to mycelium hyphae of the *T. hirsuta* strain with a longitudinal size of 1.5–2 µm. The MYC fibers were prepared by liquid-state surface fermentation and resulted in the growth of the matrix of pure mycelium hyphae. The difference in morphology and composition of the substrates was confirmed with electron microscopy and FTIR analysis. Additionally, electron microscopy demonstrated that the adsorption of PCM occurred mostly on the fiber surface in the case of MFC fibers, whereas the bundles of MYC fibers also captured the bulk PCMs within their pores in MFC–MYC and MYC composites. The successful adsorption of PCMs was additionally confirmed with FTIR spectroscopy. The difference in fiber size and morphology resulted in different poured density of the prepared phase-change composites. Compared to MFC-based composites, the poured density of the composites prepared with MFC–MYC fibers increased by 5–10 times, whereas the poured density of the composites prepared with MYC fibers increased by 10–20 times.

The DSC measurements revealed that the composites prepared with stearic acid had the latent heat storage capacity of 95–105 J/g corresponding to the actual SA content of 55–59% and the loading efficiency of 92–98%. In turn, the composites prepared with eicosane demonstrated the latent heat storage capacity of 134–138 J/g corresponding to the actual eicosane content of 57–59% and the loading efficiency of 97–98%. The actual PCM content in the composites is in good agreement with the initially added mass fraction of PCMs (60%). The cyclic DSC measurements did not reveal the substantial changes in the latent heat storage properties after 20 iterative heating/cooling cycles. However, the composites prepared with MYC fibers demonstrated a prominent shift in the shape and position of endothermic peaks due to the high content of PCMs that were melted and crystallized not on the hyphae surface. The presence of bulk PCMs captured by MYC fibers was also confirmed by TGA measurements, demonstrating the increase in thermal stability of the PCMs in the composite structure as the content of the MYC fibers increased.

Finally, the leakage test demonstrated the good shape stability of the composites prepared by adsorption of PCMs on the MFC surface with only a minor PCM leakage of 3–6% after 10 h of heating. This confirmed that the support/PCM ratio of 40/60 wt% is optimal in preparation of the shape-stable composites by adsorption of SA and eicosane on the MFC. On the other hand, the composites prepared by adsorption of SA and eicosane on MFC–MYC and MYC fibers demonstrated higher mass loss due to the leakage of the bulk PCMs from their structure. Furthermore, the most prominent mass loss was found in MYC-based composites. This demonstrated that the support/PCM ratio should be adjusted in MFC–MYC and MYC-based composites to avoid the formation of bulk PCM fraction in their structure and to promote the PCM adsorption exclusively on the fiber surface. According to our calculations, the proper support/PCM ratio is expected to be about 70/30 wt% for MYC-based composites and about 50/50 wt% for MFC–MYC-based composites. Compared to MFC-based composites, the reduction in PCM content will reduce the latent heat capacity. On the other hand, these composites demonstrated a much higher poured density that will allowx a higher mass fraction of the composites to be embedded. From this point, the MFC–MYC fibers appear to be reasonable porous support, combining high poured density, latent heat capacity, and good shape stability of the resulting phase-change composites.

## Figures and Tables

**Table 1:** Phase-change composites.

Support Type	PCM	
	Stearic Acid	Eicosane
MFC	MFC/SA	MFC/E
MFC–MYC	MFC–MYC/SA	MFC–MYC/E
MYC	MYC/SA	MYC/E

**Table 2:** Phase transition points, melting and freezing enthalpies of bare PCMs and phase-change composites, and actual PCM content calculated from DSC.

Sample	1st Cycle					20th Cycle				
	T_M_, °C	ΔH_M_, J/g	T_C_, °C	ΔH_C_, J/g	E_a_, %	T_M_, °C	ΔH_M_, J/g	T_C_, °C	ΔH_C_, J/g	E_a_, %
SA	61	178	51	182	-	-	-	-	-	-
MFC/SA	63	105	48	106	59	63	105	48	107	59
MFC–MYC/SA	64	106	48	109	59	63	103	48	105	58
MYC/SA	68	95	44	97	55	67	91	44	93	53
Eicosane	41	236	28	236	-	-	-	-	-	-
MFC/E	45	134	27	137	57	44	136	28	137	58
MFC–MYC/E	45	138	26	142	59	45	137	27	140	59
MYC/E	48	136	30	139	58	41	139	29	141	59

## Data Availability

Data are contained within the article.
